# The Structural Effects of Modality on the Rise of Symbolic Language: A Rebuttal of Evolutionary Accounts and a Laboratory Demonstration

**DOI:** 10.3389/fpsyg.2018.02300

**Published:** 2018-11-28

**Authors:** Victor J. Boucher, Annie C. Gilbert, Antonin Rossier-Bisaillon

**Affiliations:** ^1^Laboratoire de Sciences Phonétiques, Département de Linguistique, Université de Montréal, Montreal, QC, Canada; ^2^School of Communication Sciences and Disorders, McGill University, Montreal, QC, Canada; ^3^Centre for Research on Brain, Language and Music, Montreal, QC, Canada

**Keywords:** language evolution, symbolic communication, neurophysiology of speech, language development, comparative physiology

## Abstract

Why does symbolic communication in humans develop primarily in an oral medium, and how do theories of language origin explain this? Non-human primates, despite their ability to learn and use symbolic signs, do not develop symbols as in oral language. This partly owes to the lack of a direct cortico-motoneuron control of vocalizations in these species compared to humans. Yet such modality-related factors that can impinge on the rise of symbolic language are interpreted differently in two types of evolutionary storylines. (1) Some theories posit that symbolic language originated in a gestural modality, as in “sign languages.” However, this overlooks work on emerging sign and spoken languages showing that gestures and speech shape signs differently. (2) In modality-dependent theories, some emphasize the role of iconic sounds, though these lack the efficiency of arbitrary symbols. Other theorists suggest that ontogenesis serves to identify human-specific mechanisms underlying an evolutionary shift from pitch varying to orally modulated vocalizations (babble). This shift creates numerous oral features that can support efficient symbolic associations. We illustrate this principle using a sound-picture association task with 40 learners who hear words in an unfamiliar language (Mandarin) with and without a filtering of oral features. Symbolic associations arise more rapidly and accurately for sounds containing oral features compared to sounds bearing only pitch features, an effect also reported in experiments with infants. The results imply that, beyond a competence to learn and use symbols, the rise of symbolic language rests on the types of signs that a modality of expression affords.

## Introduction

There is a vast literature on the origin of spoken language, much of which offers diverging viewpoints with few areas of consensus. For instance, there is no agreement in this literature on how to define “language” (Christiansen and Kirby, 2003). On the other hand, it is widely accepted that a fundamental feature of language is its symbolic function and that, aside from humans, no other species have developed systems of signs such as those that appear in spoken language. Indeed, for some, humans are the *symbolic species* (Deacon, 1997). However, claiming the human specificity of symbolic communication rests on how one defines symbols, and the processes by which they evolved. The present paper aims at clarifying these processes within evolutionary theories while offering a demonstration of how the ability to articulate sounds presents an essential factor in the rise of symbolic language.

For some readers, this ability relating to a specific modality of expression may seem to be an obvious factor in the rise of symbolic communication. Yet general definitions of symbols often overlook processes of expression and how they contribute to the formation of signs. In fact, many evolutionists refer to Saussure (1916/1966) and Peirce (1998) and define “symbols” principally as arbitrary associations between signals and concepts of objects or events (i.e., “referents”). Authors also recognize that symbolic associations can operate from memory, when designated referents are not in the context of communication, a feature that Hockett (1960) called “displacement.” These criteria are useful in distinguishing symbols from signs that operate as *icons* or *indices*. The latter involve a *non-arbitrary* resemblance or physical connections to referents, whereas nothing in the attributes of symbols provides a clue as to their interpretation (Deacon, 2012, p. 14; Stjernfelt, 2012). However, definitions from Saussure and Pierce which focus only on arbitrary association can lead to a conceptualization of symbols as mental constructs, unrelated to modalities of expression. Indeed, Saussure saw language as reflecting a separate mental capacity or “faculty” that could generate symbols in *any* modality such as speech or gestures (Saussure, 1916/1966, pp. 10–11). Such ideas have had a lasting influence, especially on linguistic theory, where language is seen to reflect a mental competence that has little to do with modalities of performance (Chomsky, 1965; Hauser et al., 2002). But if this is the case, then why is it that symbolic language develops primarily in a vocal medium?

In focusing on this question, the following discussion draws attention to a body of work in primatology which has failed to uncover a distinct mental ability that could account for symbolic language in humans. On the other hand, we outline that humans are the only primates that possess a cortical control of vocal signals so that, overall, the data undermine the belief that symbolic language arose from an amodal mental competence. A review of this belief that underlies popular theories of the origin of spoken language serves as background to a demonstration of an opposing modality-dependent principle where symbolic language is seen as relating to an ability to articulate sounds. Such a demonstration reflects the approach of a group of studies where evolutionary scenarios are submitted to critical laboratory experiments and computer simulations (as in Gasser, 2004; Monaghan and Christiansen, 2006; Oudeyer and Kaplan, 2006; Williams et al., 2008; Monaghan et al., 2011, 2012, 2014).

### Cognitive Skills as Insufficient Factors in the Rise of Symbolic Communication

In reviewing hypotheses of the origin of spoken language, it is important to acknowledge that several cognitive abilities and neural processes which were thought to underlie symbolic communication in humans have since been observed in other primates. In particular, it has been established that, with training, apes can learn vast sets of visual symbols and can combine these productively (e.g., Savage-Rumbaugh et al., 1993; Savage-Rumbaugh and Lewin, 1996; Lyn and Savage-Rumbaugh, 2000; Savage-Rumbaugh and Fields, 2000). Follow-up studies have documented that chimpanzees and bonobos raised in symbol-rich environments can develop a vocabulary and utterance complexity similar to those of 3 year-old children (Lieberman, 1984, chapter 10; Gardner and Gardner, 1985, 1969; Pedersen, 2012). There are also reported cases where chimpanzees acquired elements of *American Sign Language* (ASL) only by communicating with other ASL-trained chimpanzees (Gardner and Gardner, 1985). Moreover, brain-imaging research indicates that associative memory in symbol learning involves similar neurological structures in apes, monkeys, and humans (e.g., Squire and Zola, 1996; Eichenbaum and Cohen, 2001; Wirth et al., 2003).

Other symbol-related abilities extend to non-human primates despite continuing claims to the contrary. Of note, the capacity to create hierarchical or embedded combinations of signs – said to reflect a process of “recursion” – was held to be uniquely human (Hauser et al., 2002; Fitch et al., 2005; cf. Hauser, 2016). Some also maintained that a related ability to combine symbols based on conceptual relations, a property termed “Merge,” was distinctly human (Chomsky, 1972, 2012; Bolhuis et al., 2012; cf. Lieberman, 2016). However, Perruchet and Rey (2005) demonstrated that chimpanzees can learn to generate embedded sequences of given symbols (and see Perruchet et al., 2004, 2006; Sonnweber et al., 2015). Additionally, research has shown that monkeys can distinguish acoustic cues in speech (as discussed by Belin, 2006), and manifest a “statistical learning” of speech sounds (Hauser et al., 2001). Several reports have further shown that non-human primates can process combinations of symbols based on conceptual relations (contra, e.g., Jackendoff, 2002; Pinker and Jackendoff, 2005; Clark, 2012; Hauser et al., 2014; Arbib, 2015). Thus, seminal work by Savage-Rumbaugh et al. (1986, 2001; Savage-Rumbaugh and Rumbaugh, 1978) revealed that training chimpanzees on paired symbols designating items and actions (“drink” and “liquids” vs. “give” and “solid foods”) facilitated the learning of combinations of novel signs. In other words, the individuals more easily acquired pairs where action symbols correctly matched signs for types of foods, which implies a processing of signs in terms of their conceptual relations (Deacon, 1997, p. 86). More recently, Livingstone et al. (2014) trained rhesus monkeys on symbols representing distinct numbers of drops of liquid (implying a coding of magnitude). On tests involving combinations of these learned symbols, the individuals not only showed a capacity to process the *relative* values of signs within a context, but also transferred these subjective valuations to new symbols, suggesting a capacity to process combined signs in terms of novel relations. Livingstone et al. (2010, 2014) also outlined that value coding of signs involves similar neural processes in humans and monkeys implicating dopamine neurons and interactions between the midbrain, the orbitofrontal cortex, and nucleus accumbens (for a critical review of other findings of this type, see Núñez, 2017).

Finally, it was also believed that only humans have the ability to *imitate*, while apes *emulate* behaviors (e.g.,Tomasello, 1990, 1996; Heyes, 1993; Fitch, 2010). Emulation has been characterized as entailing a learning of the effects of actions, rather than a copying of bodily motions. A limited capacity to imitate was thought to hinder the cultural transmission of communicative signs and tool use. Even so, some studies have shown that non-human primates can mimic the actions of their conspecifics and also learn to produce their calls and symbols (Sutton et al., 1973; Gardner and Gardner, 1985, 1969; Pedersen, 2012). Recent research has made it clear that apes can selectively apply a range of social learning processes. This includes *deferred imitation* as well as the ability to follow eye-gaze and direct attention by eye-gaze and pointing (for a review, see Whiten et al., 2009). Other related claims to the effect that only humans have “shared intentionality” and an advanced “theory of mind” (e.g., Pinker and Jackendoff, 2005) have been questioned in studies of apes reared by humans (Bulloch et al., 2008; Leavens et al., 2009; see also contra Penn and Povinelli, 2007; Call and Tomasello, 2008).

In short, research in the last decades has revealed that, contrary to held assumptions, non-human primates possess mental abilities that serve to learn and process symbols. But the fact remains that monkeys and apes in the wild do not develop repertoires of symbolic signs of the type used in spoken language (e.g., Pika et al., 2005). To illustrate the kinds of signs that arise in non-human primates, one can consider the often cited case of vervet monkeys who use distinct signals to communicate the presence of different predators (Seyfarth et al., 1980; Cheney and Seyfarth, 1990; Price et al., 2015; for similar referent-specific signals in apes, see Crockford and Boesch, 2003). It has been argued that these signs are symbols based on their seeming arbitrariness (Price et al., 2015), though some critics reject this interpretation (Hauser et al., 2002; Deacon, 2012). In fact, vocal signs of apes and monkeys appear to be largely indexical in that they reflect reactions to referents in the signaling context (see also Crockford and Boesch, 2003; Hobaiter and Byrne, 2012; Cäsar et al., 2013). Thus, research bears out that, while monkeys, apes, and humans share the cognitive abilities that are required to learn and use signs, only humans develop vast systems of symbols, and they do so primarily in a vocal medium. One implication of these results is that, even though cognitive abilities can be essential prerequisites in acquiring and manipulating symbols, some other capacity is needed to account for the emergence of these types of signs in vocal communication.

### The Role of the Medium in the Rise of Symbolic Communication

#### The Case Against Modality-Independent Accounts: “Sign Languages” and Storylines of the Gestural Origin of Language

Compared to cognitive skills, the capacity to articulate vocal patterns stands as an obvious human-specific trait. Yet in the literature on language origin, many researchers are guided by the belief that language emerged in a gestural medium. Several findings have motivated this view, which implies a conceptualization of “language” as an amodal function. One pivotal finding relates to the lack of voluntary control of vocalization in nonhuman primates.

In particular, studies by Jürgens (1976, 1992) showed that the brains of monkeys and apes lack monosynaptic fibers linking the motor cortex to laryngeal-muscle motoneurons in the nucleus ambiguous. Such findings concur with the poor control over reactive vocalizations in these species (Jürgens, 2002, pp. 242–245; Simonyan and Horwitz, 2011; Simonyan, 2014). The nervous systems of monkeys and apes do, however, present direct monosynaptic projections to motoneurons associated with the control of finger muscles, and to jaw- and lip-muscle motoneurons in the trigeminal and facial nuclei (Jürgens, 2002; Sasaki et al., 2004; Lemon, 2008). Compared to humans, though, there are fewer direct connections to tongue-muscle motoneurons in the hypoglossal nucleus (Kuypers, 1958; Jürgens, 2002), which accords with the paucity of oral segmentations or syllable-like patterns in the calls of non-human primates (Lieberman, 1968, 2006a; Lieberman et al., 1992). Taken together, these observations may have led many to believe that, since apes and monkeys produce vocal signals as inflexible reactions, symbolic language instead evolved from controllable hand gestures (e.g., Hewes, 1996; Corballis, 2002, 2009, 2017; Tomasello and Zuberbühler, 2002; Arbib, 2005, 2012, 2015; Gentilucci and Corballis, 2006; Pollick and de Waal, 2007; Arbib et al., 2008). This held belief, however, conflicts with the general observation that gestural signs of non-human primates do not function *symbolically* (and are mostly iconic or indexical). Thus, theories of the gestural origin of language are not supported by observations of extant species. Instead, the theories refer to *indirect* evidence seen to support storylines which essentially suggest that the last common ancestor (LCA) of the *homo* and *pan* (chimpanzee and bonobo) genera had, at some point, developed *symbolic gestures* from which spoken language evolved. This view, popularized by authors like Corballis and Arbib (as outlined below), has been criticized on fundamental grounds.

One objection bears on the theoretical significance given to “sign languages.” Proponents of gestural theories frequently refer to sign languages as illustrating the possibility of a gestural stage in language evolution (e.g., Corballis, 2002; Arbib, 2012, chapter 6). As such, this interpretation adheres to a view of language as deriving from an amodal faculty (e.g., Chomsky, 1965, 2000, 2006; and also Pinker, 1994; Hauser et al., 2002; Hauser, 2016). For example, gestural signs are seen to support the idea that “the language faculty is not tied to specific sensory modalities” (Chomsky, 2000, p. 121), and that “discoveries about sign languages […] provide substantial evidence that externalization is modality independent” (Chomsky, 2006, p. 22). However, such claims repeatedly disregard the fact that there is no known case where a community of normal hearers develops a sign language as a primary means of communication (Emmorey, 2005). Said differently, gestural signs as a primary system of communication generally appear where people share a pathology affecting the hearing modality – which hardly supports the notion of an amodal language capacity. On the contrary, it suggests that, given the normal human ability to control *both* vocalization and hand gestures, symbolic communication links to a vocal-auditory modality with visual gestures having an accessory imagistic role (McNeill, 2005). It follows that an account of the rise of spoken language requires an explanation of how and why symbolic signs link to the vocal medium. But assuming the gestural origin of language leads instead to posit an evolutionary shift from gestural to vocal signs, which presents a conundrum for evolutionists.

To explain this briefly, one can refer to the theories of Corballis (1992, 2003, 2009, 2010, 2017; Gentilucci and Corballis, 2006), and Arbib (2005, 2011, 2012, 2015, 2017; Arbib et al., 2008). A critical claim of these proposals is that a left-lateralized control of hand gestures in area F5 of the monkey cortex evolved into a left-sided dominance for language, which the authors locate in Broca’s area. Corballis and Arbib also refer to research showing that mirror neurons in F5 discharge when a monkey observes hand motions in others (see, e.g., Rizzolatti and Arbib, 1998; Ferrari et al., 2005). Both see in these responses a mechanism of action understanding, and conjecture that mirror neurons played a role in the shift from a gestural to a vocal-modality of communication. On how this shift occurred, it is speculated that, when the LCA descended from trees and developed bipedalism, there was a freeing of the hands allowing the development of expressive manual signs. At first, this led to putative iconic pantomimes that became conventionalized and symbolic (at a “protosign” stage), before “protospeech” developed. However, it is difficult to find in this narrative a working mechanism that converts gestures to vocal signs. For example, Arbib recently explained that pantomimes created an “open-ended semantics” which “…provides the adaptive pressure for increased control over the vocal apparatus” (Arbib, 2015, pp. 612–613; see also 2017, p. 144). By this account, the semantics of protosigns “…establishes the machinery that can then begin to develop protospeech, perhaps initially through the utility of creating novel sounds to match degrees of freedom of manual gestures (rising pitch could represent an upward movement of the hand)” (2015, p. 613). Thus, the core explanation in this view is that *semantics* drove the evolution of vocalization, and the pairing of physiological parameters of hand control with those of articulatory, laryngeal, and respiratory systems of (proto-) speech. In this account, the example of iconic signs (of pitch increase and hand rising) hardly helps to understand how vast systems of *symbolic* signs emerged. Critics have also questioned whether any realistic model can be devised to “translate” hand motions into sequences of vocalized sounds (Hewes, 1996; MacNeilage, 2008/2010, pp. 287–288).

In weighing the above scenario, one should note that it rests on the claim that a left-sided control of hand motions in the monkey cortex overlaps mirror neurons and Broca’s area in humans. It has been reported, however, that activity in mirror neurons during the perception and production of hand motions is not left-lateralized (Aziz-Zadeh et al., 2006). More generally, one might question the *a priori* validity of theories where semantics is seen to drive the evolution of mechanisms of vocalization. Such views are not limited to gestural accounts. They extend to a variety of theories that focus on cognitive skills while overlooking modality-specific constraints and how they shape signals and signs. In this orientation, it is as if symbol systems generate from some amodal mental function.

For example, Deacon (1997, 2012) suggests an account whereby signs created by apes (and children) first appear to have an indexical function. Then, when the signs are logically combined, they become more symbolic (Deacon, 1997, chapter 5). But again, apes in natural environments do not develop symbolic communication despite a capacity to combine given signs (as per the experiments of Savage-Rumbaugh et al., among others), so the question remains: how is it that symbolic signs emerge for humans and why is this linked primarily to the vocal medium? On these questions, Deacon (1997) basically offers a circular explanation: “…language must be viewed as its own prime mover. It is the author of a co-evolved complex of adaptations arrayed around a single core semiotic innovation…” (p. 44).

Aside from core semantics or semiotic functions, some theories also submit that the evolution of symbolic communication was driven by socio-cognitive functions. For example, the theory of “interactional instinct” suggests that, in language acquisition as in language evolution, children signal their intention to do something, and “The intent becomes a symbol that the child expresses in an emotionally based interaction…” (Lee and Schumann, 2005, p. 7; Lee et al., 2009). In related proposals, constellations of mental skills including “shared intentionality,” “perspective-taking,” “comprehension” (etc.), along with “thought processes” (Ackermann et al., 2014), and “purpose” (Deacon, 2011) are evoked as driving factors (for an overview of these types of factors, see the proposal of Pleyer, 2017, and Pleyer and Winters, 2015). These accounts collectively imply what some have called “mentalistic teleological” principles (Allen, 1996/2009), which do not accord with accepted features of evolution. In particular, one feature holds that evolution reflects biological change in relation to physical aspects of the environment (see Christiansen and Chater, 2008). Accepting this, it is difficult to fathom how the evolution of anatomical structures of vocal communication would be driven by “semantics,” “thoughts,” “intentions” (etc.), without some basis in the sensory effects of physical signals and physiological processes of sign production. Indeed, if one *defines* symbols as entailing associations between concepts and signal elements, then the notion that symbolic language arose from amodal mental factors presents a contradiction in terms in that, in the absence of a modality of communication, there are no signals to which concepts can associate (but cf. the pronouncement that language has little to do with communication: Chomsky, 2002, pp. 76–77; 2012, p. 11).

#### Modality-Dependent Accounts of the Rise of Symbolic Language

##### Mimesis and procedural learning

Contrary to the above viewpoint, several authors have submitted that language first evolved in the vocal modality and that this medium imposes particular constraints on the formation of signs (e.g., Lieberman, 1984, 2000, 2003, 2007, 2016; MacNeilage, 1998, 2008/2010; Studdert-Kennedy, 1998; MacNeilage and Davis, 2000, 2015). This basically refutes claims that sign languages and spoken languages have similar structure reflecting an amodal mental capacity (e.g., Bellugi et al., 1989). Thus, MacNeilage (2008/2010, chapter 13) explained that vocal and gestural modalities shape signs quite differently: for instance, gestural signs are holistic and can involve simultaneous hand and body motions whereas, in spoken language, sounds are strictly sequential, and are constrained by articulatory and respiratory-phonatory systems. It is useful to note that such a viewpoint is echoed in recent studies of emerging sign language that explicitly focus on the link between signs and constraints on gestures. Most revealing is the work of Sandler et al. on *Al-Sayyid Bedouin Sign Language* (ABSL), which led to expressed reservations both on gestural accounts of language origin, and linguistic methods which have dominated research on sign languages.

Traditionally, the study of sign languages has relied on linguistic analyses and assumptions which focus on abstract phonological features, “word” categories and their syntax, but which entirely neglect modality-specific differences between gesture and sound production (MacNeilage, 2008/2010). Sandler et al. noted several difficulties in attempting to pigeon-hole gestures in terms of assumed linguistic features and units, and instead examined gestures as such (Sandler, 2012, 2013, 2017; Sandler et al., 2014). Their study of ABSL revealed a correspondence between developing motor aspects of gestures and the complexity of expressed concepts, which question the doctrine that spoken and gestural systems can derive from a common modality-independent function. As Sandler (2013, pp. 194–195) remarked, in commenting Arbib’s gesture theory of language origin –

…a different motor system controls language in each modality [gestural and vocal], and the relation between that system and the grammar is different as well. Considering the fundamental differences in motor systems, I am mindful of the reasoning of experts in the relation between motor control and cognition (Donald, 1991; MacNeilage, 2008/2010) who insist on the importance of the evolution of the supporting motor system in the evolution of language to the extent that ‘mental representation cannot be fully understood without consideration of activities available to the body for building such representations… [including the] dynamic characteristics of the production mechanism’ (Davis et al., 2002).

The findings also implied a reevaluation of linguistic assumptions on sign languages (Sandler, 2012, pp. 35–36):

…many believe that sign languages rapidly develop into a system that is very similar to that of spoken languages. Research charting the development of Nicaraguan Sign Language (e.g., Senghas, 1995; Kegl et al., 1999), which arose in a school beginning in the late 1970s, has convinced some linguists, such as Steven Pinker, that the language was “created in one leap when the younger children were exposed to the pidgin signing of the older children…” (Pinker, 1994: 36). This is not surprising if, as Chomsky believes, “…language evolved, and is designed, primarily as an instrument of thought, with externalization a secondary process.” (Chomsky, 2006: 22). […] Our work on ABSL, of which the present study is a part, suggests that those of us who contributed to this general picture may have overstated our case.

In sum, motor aspects of gesture and sound production shape symbol systems differently and storylines suggesting that vocal symbols developed from gestural signs bear intractable problems of gesture-to-sound conversion. But while such problems suggest a necessary link between symbolic language and vocal processes, not all modality-specific accounts deal with the rise of symbolic signs.

As an example, Lieberman’s proposal focuses on how spoken language, viewed as a combinatorial system, links to the evolution of speech processes in conjunction with the basal ganglia and its function as a “sequencing engine” (e.g., Lieberman, 2003, 2006b, 2016). However, this proposal does not specifically address the issue of how vocal symbols emerged. MacNeilage, on the other hand, views symbols as the most fundamental factor of language evolution (MacNeilage, 2008/2010, p. 137). In his “frame/content” theory, the vocal modality provided a prototypal frame, the syllable, which is seen to originate in cyclical motions such as mastication (MacNeilage, 1998, but see Boucher, 2007 who shows that cyclicity in speech articulation has little to do with mastication or chewing motions). MacNeilage argues that, for arbitrary symbols to emerge, “…hominids needed to evolve a capacity to invent expressive conventions” (p. 99), though this did not arise from a “higher-order word-making capacity” (as in Hauser et al., 2002). Instead, the capacity to form “words” rests on mimesis and procedural learning, which refines actions and memory of actions. Donald (1997) described these processes as a capacity to “…Rehearse the action, observe its consequences, remember these, then alter the form of the original act, varying one or more parameters, dictated by the memory of the consequences of the previous action, or by an idealized image of the outcome” (p. 142). MacNeilage, quoting Donald, submits that while human infants manifest procedural learning at the babbling stage, great apes do not, so that “It would be no exaggeration to say that this capacity is uniquely human, and forms the background for the whole of human culture including language” (Donald, p. 142). Yet studies of tool manufacture in chimpanzees challenge this claim.

For instance, in a recent longitudinal study, Vale et al. (2016, 2017) observed that chimpanzees are not only successful in creating tools to retrieve rewards. They can also retain a procedure of tool manufacture for years and transfer this knowledge to new tasks, indicating an ability to acquire skills. Again, procedural learning, as other cognitive abilities, is required in symbol learning. However, it is not a sufficient factor in accounting for the human-specific development of symbolic language in a vocal medium. Nonetheless, the above proposals share the view that symbolic communication does not emerge from mental functions alone but essentially links to a capacity to produce patterns like syllables and babble.

##### “Sound symbolism”: Questions of efficiency and ease of learning of iconic signs

Another theory which also rests on mimesis suggests that symbolic language may originate from iconic vocal signs (or sound symbols) on the assumption that “iconicity seems easier” than making arbitrary associations (Kita, 1997; Monaghan et al., 2012; Thompson et al., 2012; Imai and Kita, 2014; Sereno, 2014; Perlman et al., 2015; Massaro and Perlman, 2017). The idea that symbols arose from a mimicking of objects and events partly draws from experiments by Sapir (1929) and Köhler (1947) on “sound-shape” pairings, where listeners judge perceived consonants and vowels as relating, for instance, to “angular” and “rounded” forms, or Ohala’s (1994) “frequency code” where pitch is related to features like “size” and “brilliance” (e.g., Maurer et al., 2006; the frequency code also extends across species that use vocal pitch in signaling aggressive/passive intentions; Morton, 1994). The central assumption is that such iconic cues are vital to the ontogenesis and phylogenesis of language because they inherently facilitate the sound-meaning mappings and the displacement of signs (e.g., Monaghan et al., 2014). Experiments showing sound-form associations in infants and adult learners are often cited as supporting this view. For instance, a study by Walker et al. (2010) shows that pre-babbling infants of four months are able to associate pitch to features such as height and brightness (see also Monaghan et al., 2012). However, the experiments do not serve to demonstrate the facilitating effect of iconic sounds on language development, or the necessity of an iconic stage in developing arbitrary signs of oral language.

On these issues, attempts to relate iconic signs to spoken language face a logical problem. Iconic gestures or sounds offer highly restricted sets of signs compared to arbitrary symbols. Any restriction on the number of signs will inherently limit the diversity of form-referent associations and thus the *efficiency* of signs in communicating fine distinctions in meaning, leading some to see that “While sound symbolism might be useful, it could impede word learning…” (Imai and Kita, 2014, p. 3; also Monaghan and Christiansen, 2006; Monaghan et al., 2012). This is evident in signs serving to *name* referents where sound symbols are quite limited (e.g., it is difficult to conceive how one could mimic open sets of lexemes such as “proper nouns”). On the idea that vocal iconic signs may nonetheless be easier to learn than arbitrary signs, computational models and experiments involving adult learners lead to the opposite conclusion (Gasser, 2004; Monaghan et al., 2011, 2012, 2014). For instance, in a series of experiments, Monaghan et al. (2012, 2014) compared the learning of arbitrary and iconic (systematic) sign-meaning pairs taking into account given co-occurring “contextual” elements. In both neural network simulations and behavioral experiments, arbitrary form-meaning pairs were learned with fewer errors and more rapidly than iconic pairs. Still, such tests do not address the issue of how *arbitrary* signs arise in the vocal medium, and why this is specific to humans.

##### Segmented vocalization and the rise of symbolic signs: A demonstration

The aforementioned simulations and tests focus on the learning of sound-shape associations by infants and adults with respect to the perception of *given* signs provided by an experimenter. Such protocols do not address the issue of how signs emerge, and infants in the first months of life may not produce symbolic signs. However, it is well-established that maturational changes in the vocal apparatus coincide with the rise of such signs in child speech. On this development, comparisons of the vocal processes of human infants and apes are revealing of the mechanisms underlying the rise of vocal symbols. In particular, pre-babbling infants, like nonhuman primates, are obligate nasal breathers and produce sounds with nasal resonances (Negus, 1949; Crelin, 1987; Thom et al., 2006). Acoustically, nasalization dampens upper harmonics and formants, reducing the distinctiveness of sounds. Moreover, continuous nasal air-flow during vocalization implies that articulatory motions may not create salient features of oral segmentation (Lieberman, 1968, 1984; Lieberman et al., 1972; Thom et al., 2006). For instance, producing multiple elements like stops [p,t,k,b,d,g], fricatives [f,s,ʃ,v,z,Ʒ], or any articulatory pattern of segmentation requires modulations of oral pressure which are difficult to achieve in a system where air flows through the nose. For this reason, early productions of infants largely appear as continuous nasalized cries and vocalizations which, as in the vocal sounds of non-human primates, are divided by breath interruptions and glottal closures (Crelin, 1987; Lynch et al., 1995). At three months, though, humans manifest a control of pitch contours, which can reflect a refinement of monosynaptic connections between the motor-cortex and motoneurons of laryngeal muscles (Ackermann et al., 2014). Some suggest that, at this stage, vocalizations become less reactive and iconic, and can involve a symbolic coding of pitch (Oller, 2000; Oller and Griebel, 2014). Subsequent supraglottal changes occur which are also human specific.

Of interest is the progressive decoupling of the nasopharynx indexed by the distancing of the epiglottis from the velum. In human newborns, as in many mammals, the epiglottis habitually overlaps the velum creating a sealed passage that allows nasal breathing while ingesting food and extracting breast milk (Negus, 1949; Kent, 1984; Crelin, 1987). It should be noted that the decoupling of the nasopharynx involves *soft* tissues *–* hence the difficulty of dating this change from fossil records. Many works of comparative anatomy involving CT and MRI scans discuss this decoupling by reference to a “descent of the larynx” which is indexed by a lowering of the hyoid bone attached to the larynx, and related measures of vocal-tract length. Because this lowering is observed across species, some have concluded that laryngeal descent has little to do the rise of spoken language in humans (e.g., Fitch and Reby, 2001; Fitch, 2010, chapter 8). However, laryngeal descent is only accompanied by a *permanent* decoupling of the epiglottis and the velum in humans, beginning at about 6–8 months (Kent, 1984; Nishimura et al., 2006; Lieberman, 2007). Following this decoupling, articulatory motions can create a variety of salient oral features in vocal signals, which constitutes a pivotal human-specific development. Though some contend that other species can produce a range of vowel-like resonances (Fitch, 2000, 2005; Fitch and Reby, 2001), only humans segment these resonances using varying articulatory motions. This important change is accompanied by a general increase in rhythmic behavior (Iverson et al., 2007) leading to *babble* (Locke, 1995; Oller, 2000). Combining this morphological development with a capacity to modulate pitch contours confers the unique ability to manipulate both tonal and articulatory patterns. As noted, compared to other primates, humans develop direct monosynaptic projections to motoneurons of laryngeal muscles and also present a greater ratio of direct connections to tongue-muscle motoneurons which together support a control of orally modulated patterns of vocalization.

The symbolic potential of these patterns can be fathomed by considering that, in canonical babble, reduplicated syllables containing articulatory features such as [dada], [mamama], [nana] (etc.) become rapidly associated with caregivers, food, and other contextual referents. These early signs show that the *arbitrariness* underlying symbolic language can be *inherent* to the types of sounds that arise with orally segmented speech (a point also noted by Corballis, 2002), and that contextual information suffices to establish functional sound-meaning associations. In fact, the developmental literature does not suggest that iconic sounds or gestural mimics precede the rise of symbolic signs in canonical and variegated babble (Locke, 1995; Oller, 2000). On the other hand, it is generally acknowledged that the rise of vocal symbols accompanies a shift from pitch-varying to orally segmented vocalizations, though some symbol coding of pitch can precede this shift (Oller and Lynch, 1992; Oller, 2000).

Overall, the above developments suggest that the human capacity to produce orally segmented vocalizations presents a *necessary* – though not sufficient *–* factor in the rise of symbolic signs. In other words, cognitive and perceptual abilities are certainly required in forming sound-meaning associations, but these abilities are present at a basic level in non-human primates who do not develop symbolic signs like those of spoken language. Hence, the particular shift from pitch-varying to articulated vocalizations appears essential to the emergence of efficient vocal symbols in that, compared to pitch patterns, oral modulations offer a more diverse set of salient articulatory features by which to create fine distinctions in meaning.

Of course, a direct demonstration of this shift on the development of vocal symbols is not possible (i.e., one may not experimentally manipulate the human ability to articulate sounds). But one can artificially reduce acoustic features associated with articulatory motions by filtering signals so as to observe effects on the formation of symbolic associations. This guided the design of a straightforward demonstration using a task where listeners learn to associate pictures to unfamiliar speech sounds (in this case, two-syllable “words” spoken in Mandarin). In the task, sounds are presented with sets of possible referents (pictures of familiar objects) allowing the rise of associations through repeated trials. To evaluate the effect of a shift from intoned to orally articulated patterns on symbolic associations, filtered stimuli are presented bearing only pitch-varying intonations, then unfiltered stimuli with oral features are presented (for an example of the stimuli, see “Materials and methods” Figure 3). To support word-picture associations, two types of feedback are used representing two basic responses that can be obtained in a context of verbal learning. In the first type (henceforth *feedback A*), learners make an association and obtain an indication on whether or not the association is correct (“yes/no”). In the second type (*feedback B*), learners additionally receive information on what the sound designates (the correct picture is displayed). The first feedback condition emphasizes a process of inference, while the second favors rote learning. In both conditions, the prediction was that symbolic sound-meaning associations would form more rapidly for orally segmented sounds than for intoned patterns principally because of the greater efficiency of oral features in making distinctions in meaning.

## Results

**FIGURE 1:**
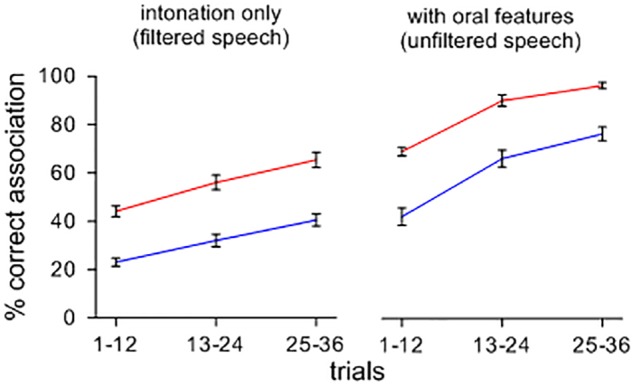
Percent correct sound-picture association for different sets of lexemes presented with and without a filtering of oral features. Note that, when intoned items are presented with their oral features, symbolic associations rise more quickly and more accurately across feedback conditions.

Figure 1 shows the effect of a change from intoned to orally modulated patterns on the rise of symbolic associations. Upon hearing sets of items containing features of oral articulation, arbitrary sound-meaning associations shift and form more rapidly and accurately than when the items are heard with only their tonal attributes, and this effect appears across feedback conditions. A repeated-measures ANOVA confirmed main effects of stimuli type [*F*(1,39) = 2.202; *p* < 0.001; η_p_^2^ = 0.822; *MSE* = 0.012], and feedback ([*F*(1,39) = 3.581; *p* < 0.001; η_p_^2^ = 0.896; *MSE* = 0.011]. Orally segmented stimuli yielded more correct associations than filtered stimuli, and *feedback B* led to more correct associations than *feedback A*. There was no significant interaction between the type of contexts and feedback conditions [*F*(1,39) = 8.740E-5; n.s.; η_p_^2^ = 0.000; *MSE* = 0.012].

**FIGURE 2:**
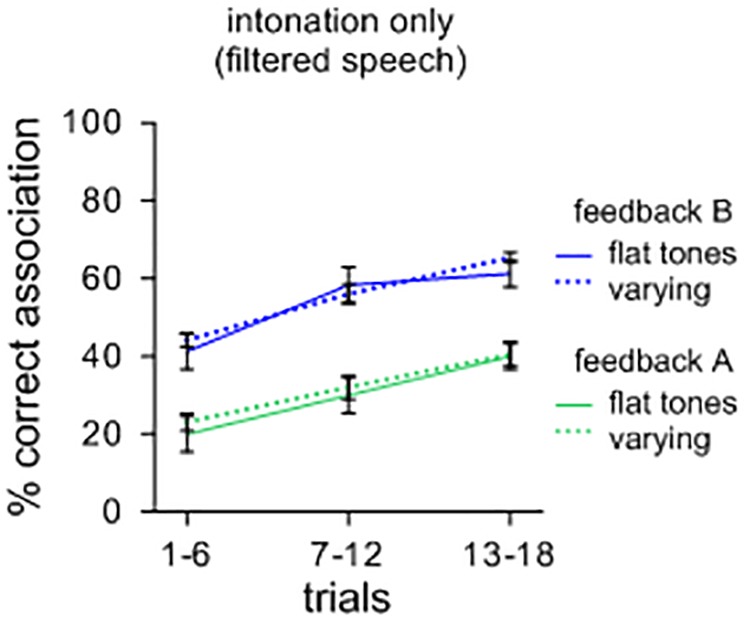
Percent correct sound-picture association for different sets of filtered lexemes. Items with varying (rising and falling) tones did not lead to more symbolic associations than items with only flat tones.

As a further illustration, a separate comparison of sound-meaning associations was performed for sets of filtered items with different tones of Mandarin. Half of the contexts had initial flat tones while the other half contained variable rising and falling tones. One could surmise that symbolic associations could be facilitated for sets of items with varying tones compared to items that have only flat tones. However, as Figure 2 illustrates, this effect did not occur. A repeated measures ANOVA showed no main effects of tonal patterns [*F*(1,39) = 0.049; n.s.; η_p_^2^ = 0.069; *MSE* = 0.017], but a main effect of feedback [*F*(1,39) = 2.175; *p* < 0.001; η_p_^2^ = 0.757; *MSE* = 0.018] with *feedback B* leading to more correct associations than *feedback A*. Again, there was no significant interaction between the types of contexts and feedback [*F*(1,39) = 0.000; n.s.; η_p_^2^ = 0.001; *MSE* = 0.013]. A visual comparison with Figure 2 suggests that, overall, intonational features are less efficient in supporting the formation of symbolic associations than oral features. It should also be weighed that many languages unlike Mandarin do not code tone at all in distinguishing lexical items, and thus, from a general perspective, intonation as such may not suffice in distinguishing large repertoires of lexemes.

## Discussion

The preceding results demonstrate a seemingly self-evident effect: beyond the competence of the above learners and their experience with language, they were more prone to form arbitrary sound-meaning associations with oral patterns of speech than when speech contained only intonational patterns. Specifically, when shifting from items with tonal features to items that include features of articulation, symbolic associations were formed more rapidly and accurately. Of course, these observations and the above test are not meant to reflect evolution. Their purpose is to demonstrate the basic point that one may not account for the rise of symbolic signs in language without some reference to the types of signals that a modality of expression affords. Yet this is not a generally accepted principle.

As seen in the above review, the idea that symbolic language evolved from modality-independent cognitive abilities is widespread. One should also remark that the definitions of symbols that guide much of the work on language evolution refer to 19th century writers like Peirce and Saussure who did not consider how processes of modality can shape signals and signs. Following this tradition, storylines of language origin are largely oriented by the belief, popularized in linguistic theory, that symbolic language derives from a mental competence or faculty which has little to do with processes of expression like gestures and speech (Saussure, 1916/1966; Chomsky, 1965). From this standpoint, the rise of symbolic language would seem “mysterious” (Hauser et al., 2014), “puzzling” (Aitchison, 2000; Bouchard, 2015), and can lead to ask “why only us” (Berwick and Chomsky, 2015) or to speculations of evolutionary saltations (e.g., Hauser et al., 2002). However, linguistic analyses that focus on abstract categories and units overlook the structural effects of varying modalities of expression. On these effects, we noted that work on emerging sign and spoken language bears out that motor aspects of gestures and vocalization do shape sign systems very differently. As for the idea that symbols arise from a mental capacity, decades of research has shown that monkeys and apes have a basic competence to learn, process, and combine symbolic signs. Yet they do not develop the types of productive vocal symbols found in spoken language. One implication is that the human specificity of symbolic language may not be explained in terms of cognitive capacities alone. Other factors relating to the human ability to control vocal signals are needed to account for symbolic language and why it arises primarily in oral medium.

On these factors, several works of comparative physiology have identified human-specific changes that can underlie the rise of symbolic signs, and these reflect in ontogenesis in terms of a shift from pitch-varying patterns to orally articulated babble. As Corballis (2002), pp. 187–188) indicated, articulatory modulations of sounds generate numerous features in signals creating inherently arbitrary signs. These signs can be rapidly associated with co-occurring referents in the course of developing language. No stage of iconic gestures or “sound-symbolism” appears to precede this development (Locke, 1995; Oller, 2000). But nor does such a stage seem necessary. In fact, several studies have shown that infants, even pre-babblers, readily associate heard “words” with referents, and use these symbols to categorize objects or parts of objects (see Fulkerson and Waxman, 2007; Ferry et al., 2010, and for children of 12 months, MacKenzie et al., 2011). Interestingly, the reports also show that infants are less successful in forming symbolic associations when presented with sounds like intoned [mmm] or sounds that cannot be articulated, and instead attend to familiar speech (MacKenzie et al., 2011; Marno et al., 2015, 2016; and for evidence that activity in language areas of the brain are organized in terms of speech sounds, see Magrassi et al., 2015). This ability to acquire symbols is not distinctly human, as we noted, but communication by way of orally articulated signs is. One can only speculate on what might have evolved had humans been limited to pitch-varying calls. From the above results, it can be surmised that pitch-controlled signals without features of oral articulation would restrict the rapid formation of efficient (accurate) symbols. On the phylogenesis of this capacity, certainly a pivotal factor is the decoupling of the nasopharynx which contributed to free the oral tract allowing an articulatory modulation of sounds (e.g., Negus, 1949; Lieberman, 1984, 2006b; Crelin, 1987). Some see this as a consequence of bipedalism (originating 5–7 million years ago) and that bipedalism further led to a decoupling of respiration and locomotion that supported an independent control of phonation (Provine, 2005; see also Maclarnon and Hewitt, 1999, 2004). However, as we mentioned, bone markers in fossil records may not serve to index the separation of the nasopharynx, so dating this change as co-evolving with bipedalism appears problematic.

## Materials and Methods

### Participants

The participants were 40 speakers aged 20 to 41 years (mean of 25 years; 19 females) with no history of hearing problems or knowledge of any tone language. Prior to the task, all were screened for normal memory performance using a standard digit-span test (Wechsler, 1997).

### Speech and Picture Stimuli

The original speech stimuli consisted of 24 two-syllable Mandarin lexical items (“words”) that matched 24 black-and-white pictures of objects (Snodgrass and Vanderwart, 1980). Half of these contexts had an initial flat tone and half a varying rising or falling tone (used to compare effects of coded pitch). The items were produced in isolation by a native female speaker of Mandarin and recorded using a headset microphone (AKG, model C-477-WR) and an external sound card (Shure, model X2u) set to a 16-bit resolution and 44.1 kHz sampling rate. The recorded items were stored as separate .wav files, and copies of these files were low-passed filtered at 350 Hz using an IIR function (*Goldwave*, v. 6.19) giving 24 filtered and 24 unfiltered versions of each of the lexemes. Finally, all of the stimuli were amplitude normalized to obtain similar dB intensities.

**FIGURE 3:**
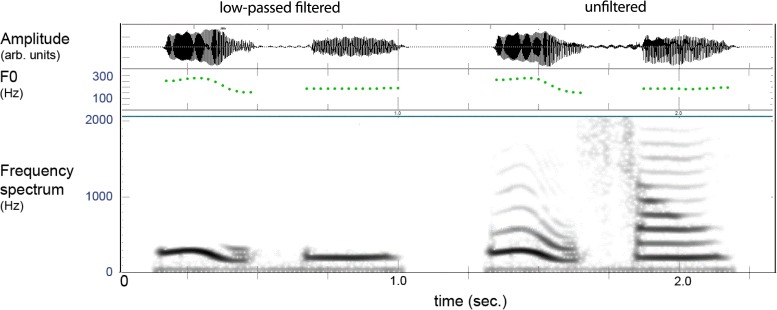
Example of filtered (left) and unfiltered (right) versions of a two-syllable lexeme in Mandarin. The filtering leaves the pitch (F0) of the lower harmonic, and the item is heard as vocalized tones without “vowel” or “consonant” features.

Figure 3 provides an example of the filtered and unfiltered version of a spoken lexeme where one can see that only the lower harmonic is present in the filtered version, which gave natural-sounding pitch patterns with a vocalic quality. The distinctiveness of these contexts was evaluated by an external judge (not the authors) using a discrimination test. This was done by creating duplicates (*AA*) of the filtered items and placing another filtered test item (*X*) with similar tones before and following the pair, giving series of *AAX* and *XAA*. All tokens *X* were correctly discriminated indicating that the filtered stimuli retained distinguishable elements.

### Test Design and Procedure

A repeated measures design was used with a counterbalancing of blocks of spoken lexemes on two type conditions (filtered vs. unfiltered), and two feedback conditions (*A* vs. *B*, described earlier), while taking into account a specific order of presentation (filtered items with *feedback A* were presented before filtered items with *feedback B*; then, unfiltered items with *feedback A* were presented before unfiltered items with *feedback B*). The latter presentation sequence basically aimed to capture effects of a shift from tonal to orally segmented patterns. The 24 lexemes were arranged into four lists of trials, each containing six repetitions of six different lexemes, giving 36 test trials per list. Each trial lexeme within the lists was matched to an array of four pictures with one correct lexeme-picture match and three random fillers. The counterbalance design implied that, across participants, all lists were presented an equal number of times in each condition.

In the experiment, a test trial began with a three-second display of four pictures, followed by a heard speech stimulus. The participant then had to select a likely speech-picture match by pressing a number on a keyboard (a forced-choice task). After the participant’s key-press response, a feedback slide was displayed for two seconds. During the test, participants sat in a sound-treated room in front of a monitor and listened with headphones (Beyerdynamic, model DT 250) to the speech stimuli which were delivered via software (*E-Prime 2*). The sound stimuli had peak values of 71 dBA at the ears, as measured with a sound-level meter and headphone adapter (Digital Recordings, model DR-1). Practice runs were provided prior to the test, which proceeded without interruption. Statistical analyses of the responses used the procedures of *SPSS* (v. 17.0).

## Ethics Statement

This study was carried out in accordance with the recommendations of the ‘Declaration of Helsinki’ with written informed consent from all subjects. The protocol was approved by the Comité d’Éthique de la Recherche de la Faculté d’Arts et Sciences de l’Université de Montréal.

## Author Contributions

VB conceived the theoretical arguments and, with AG, designed the test. AR-B contributed to the data acquisition and procedure. All three authors contributed to the data analysis, interpretation, critical revision, and final approval of the paper.

## Conflict of Interest Statement

The authors declare that the research was conducted in the absence of any commercial or financial relationships that could be construed as a potential conflict of interest.
